# Clinical remission among severe asthmatics on monoclonal antibody therapy: real-world outcomes at 2 years

**DOI:** 10.1183/23120541.00261-2024

**Published:** 2024-12-16

**Authors:** Fred Fyles, Rachel Burton, Amy Nuttall, Hannah Joplin, Laura Watkins, Hassan Burhan

**Affiliations:** 1 Liverpool University Hospitals NHS Foundation Trust, Liverpool, UK; 2 Liverpool School of Tropical Medicine, Liverpool, UK; 3 University of Liverpool, Liverpool, UK

## Abstract

Severe asthma is a chronic inflammatory condition characterised by recurrent exacerbations requiring oral corticosteroids (OCS), despite optimal use of preventer therapy [1, 2]. Monoclonal antibody (MAb) therapies targeting immunological pathways implicated in severe asthma pathogenesis may reduce maintenance OCS (mOCS) use and annualised exacerbation rates (AERs). Recently, there has been a shift toward clinical remission being a treatment goal in patients with severe asthma on MAb therapy [3] – while there is no set definition of clinical remission, it has been suggested that it include no OCS use, lung function stabilisation and minimal symptom burden [4].

## Shareable abstract

**Real-world data suggest clinical remission is a feasible treatment goal of monoclonal antibody therapy. Evidence of ongoing response post-12 months may be used to inform treatment decisions. Further work is needed to standardise criteria for remission.** https://bit.ly/4cA6TkG

*To the Editor:*

Severe asthma is a chronic inflammatory condition characterised by recurrent exacerbations requiring oral corticosteroids (OCS), despite optimal use of preventer therapy [1, 2]. Monoclonal antibody (MAb) therapies targeting immunological pathways implicated in severe asthma pathogenesis may reduce maintenance OCS (mOCS) use and annualised exacerbation rates (AERs). Recently, there has been a shift toward clinical remission being a treatment goal in patients with severe asthma on MAb therapy [3] – while there is no set definition of clinical remission, it has been suggested that it include no OCS use, lung function stabilisation and minimal symptom burden [4]. While clinical studies [5–12] report remission rates of 21.7–68.5% in patients on MAb therapy, available real-world data on long-term remission past 12 months are lacking.

We report a retrospective data analysis of patients with physician-confirmed severe asthma completing ≥24 months of MAb therapy. We aimed to investigate: clinical response to MAb therapies at 12 and 24 months; clinical remission rate at 12 and 24 months, defined as no use of mOCS, AER 0, and Asthma Control Questionnaire (ACQ-6) score ≤1.5; and predictive factors for “early” clinical remission (by 12 months) and “late” clinical remission (by 24 months).

Data were collected on patients who began MAb therapy between August 2013 and December 2021, and who completed ≥24 months’ therapy. The data collected included: baseline characteristics (sex, body mass index (BMI), comorbidities, smoking status and medication use) and clinical outcomes (mOCS, AER, blood eosinophil count (BEC), exhaled nitric oxide fraction (*F*_ENO_), ACQ-6 score and Asthma Quality of Life Questionnaire (AQLQ) score) at baseline, 12 months (T1) and 24 months (T2).

Overall, 485 patients were started on MAb therapy between August 2013 and December 2021, of whom 282 (58.1%) completed ≥24 months’ therapy and were included in our cohort (mean±sd age 52.1±15.7 years, BMI 32.8±7.7 kg·m^−2^; 62% female). All patients were on high-dose inhaled corticosteroid with at least one additional controller therapy. 122 (43.2%) were on mOCS at baseline and average AER was 5.6±3.2. The most common comorbidities were allergic rhinitis (53%) and nasal polyposis (23%). The majority were on anti-interleukin-5 agents, with mepolizumab and benralizumab making up 32% and 51% of MAb choices respectively.

Across the entire cohort there was statistically significant improvement from baseline to T1 and T2 in mOCS, AER, BEC, ACQ-6 and AQLQ. There was also statistically significant improvement in mOCS from T1 to T2, suggesting ongoing clinical response beyond 12 months (table 1).

**TABLE 1:** Clinical outcomes of entire cohort (n=282)

	Baseline	T1	T2
**mOCS, mg·day^−1^ (mean±sd)**	3.9±6.1	2.0±4.3*	1.5±4.2^#^^,¶^
**AER (mean±sd)**	5.6±3.2	1.7±2.1*	1.9±2.3^¶^
**BEC, ×10^9^ per dL (mean±sd)**	0.43±0.43	0.09±0.34*	0.10±0.25^¶^
**FEV_1_, L (mean±sd)**	2.06±0.80	2.15±0.86*	2.10±0.84^#^
**FEV_1_, % of predicted (mean±sd)**	74.1±20.7	77.0±22.1*	76.1±22.1
***F*_ENO_, ppb (mean±sd)**	51.9±47.1	51.9±45.6	48.0±47.5^¶^
**ACQ-6 score (mean±sd)**	3.59±1.32	2.44±1.50*	2.57±1.54^¶^
**AQLQ score (mean±sd)**	2.94±1.27	4.28±3.81*	3.73±1.57^#^^,¶^
**Remission criteria**			
No mOCS	161 (57%)	210 (74.5%)	226 (80.1%)
AER 0	14 (5%)	103 (36.5%)	104 (36.9%)
ACQ-6 score ≤1.5	11 (4%)	60 (21.3%)	46 (16.3%)
Clinical remission	0 (0%)	31 (11.0%)	27 (9.6%)
Omalizumab (n=41)		4	3
Mepolizumab (n=89)		7	5
Benralizumab (n=145)		20	18
Reslizumab (n=2)		0	1
Dupilumab (n=5)		0	0

T1: 12 months; T2: 24 months; mOCS: maintenance oral corticosteroids; AER: annualised exacerbation rates; BEC: blood eosinophil count; FEV_1_: forced expiratory volume in 1 s; *F*_ENO_: fractional exhaled nitric oxide; ACQ-6: Asthma Control Questionnaire; AQLQ: Asthma Quality of Life Questionnaire. *: p<0.05 from baseline to T1; ^#^: p<0.05 from T1 to T2; ^¶^: p<0.05 from baseline to T2.

Applying criteria for clinical remission, at T1, 210 (74.5%) had no mOCS, 103 (36.5%) had AER 0 and 60 (21.3%) had ACQ-6 score ≤1.5. 31 (11.0%) patients met all three criteria and demonstrated clinical remission (four on omalizumab, seven on mepolizumab and 20 on benralizumab). Patients who demonstrated clinical remission at T1 had a lower baseline mOCS use (1.66±4.34 *versus* 4.13±6.23 mg·day^−1^, p<0.05) and ACQ-6 score (2.90±1.41 *versus* 3.68±1.29, p<0.05) compared to those who did not; other baseline clinical parameters were not significantly different between the groups.

At T2, 226 (80.1%) had no mOCS, 104 (36.9%) had AER 0 and 46 (16.3%) had ACQ-6 score ≤1.5. 27 patients (9.6%) demonstrated clinical remission at T2 (three on omalizumab, five on mepolizmab, 18 on benralizumab and one on reslizumab). Patients who demonstrated clinical remission at T2 had a significantly lower baseline mOCS use (1.81±3.63 *versus* 4.08±6.26 mg·day^−1^, p<0.05) and ACQ-6 score (3.09±1.31 *versus* 3.65±1.32, p<0.05) than those who did not. Similarly, patients who demonstrated remission at T2 had improved mOCS use (0.54±1.67 *versus* 2.14±4.44 mg·day^−1^, p<0.05), AER (0.30±0.47 *versus* 1.89±2.20, p<0.05), ACQ-6 score (0.83±0.87 *versus* 2.63±1.42, p<0.05) and AQLQ score (5.69±1.05 *versus* 4.13±3.96, p<0.05) at T1 compared to those who did not.

In terms of relative improvement, patients who demonstrated clinical remission at T1 and T2 had significantly greater improvements from baseline in AER, ACQ-6 and AQLQ than those who did not. However, there was no significant difference in reduction in mOCS from baseline to T1 or T2 among those demonstrating clinical remission.

These findings, among a real-world clinical cohort, demonstrate a proportion of severe asthma patients on MAb therapy at our centre were able to achieve clinical remission, and as such it could form a realistic treatment goal, in line with previously published literature. Our analysis demonstrates significant ongoing improvement in mOCS beyond 12 months, suggesting that, for our cohort, a partial MAb response may continue to improve in the future. This message is reinforced by the fact that 5% of patients demonstrated clinical remission only after 24 months’ therapy; this will impact our centre's decisions on whether to continue on MAb treatment beyond 12 months in patients whose response has been partial.

Our analysis reported a lower remission rate than existing literature. Within some of these studies – for example, the pooled *post hoc* analysis of the SIROCCO, CALIMA and ZONDA trials by Menzies-Gow *et al.* [8] – the patient cohort is likely to be markedly less heterogenous than our own. Within other real-world studies, there has been marked variation in the definition of clinical remission, along with variations in study definitions (*e.g.* differentiation between exacerbation definitions), which makes direct comparisons challenging. As previously raised [13, 14], this highlights the need for a unified definition of clinical remission for use in both clinical research and practice in the future. Our analysis suggested baseline mOCS use and ACQ-6 score may be a predictor of future clinical remission development, with patients whose asthma was better controlled at treatment initiation being more likely to develop clinical remission. This is in line with previously published data suggesting low ACQ-6 and mOCS use are associated with an increased clinical remission rate [6, 7, 10].

While the majority of our patients demonstrated significant reduction in mOCS and AER, a smaller proportion demonstrated the improvement in ACQ-6 score required for our definition of remission. It may be that this reflects the fluctuant nature of asthma symptoms, which can vary depending on factors such as season or weather, and suggests the use of a patient-reported outcome measure with a short recall period, such as the ACQ-6, may not reflect overall clinical improvements over long periods of time. Further patient-centred work may be required to incorporate validated patient-reported outcome measures within a clinical remission definition.

Given the study's retrospective nature, some data were unavailable across the cohort, including age of onset of asthma symptoms. Furthermore, as this was a single-centre study, these findings may not be generalisable outside our practice. A key limitation of the study, other than its retrospective, single-centre nature, is the shifting clinical landscape over which these patients were treated: the first patients included within this study were started on MAb therapy at a time when only omalizumab (under the brand Xolair) was clinically available in the UK for treatment of severe asthma, which may have impacted decisions on continuation of treatment. Furthermore, due to the interruption to clinical services necessitated by the coronavirus pandemic after March 2020, some datasets for patients were incomplete; most notably, due to its classification as an “aerosol-generating procedure”, pulmonary function testing was not available for all patients; as such, we did not include improvement in lung function as a criterion for our definition of clinical remission.

In conclusion, we demonstrated a proportion of real-world patients with severe asthma in our cohort treated with MAb therapy will demonstrate clinical remission by 24 months. Baseline mOCS use and ACQ-6 score were key predictors for which patients will go on to develop clinical remission. There is evidence of sustained improvement in response to MAb therapy beyond 12 months, with a proportion of patients demonstrating clinical remission at 24 months, which may influence clinical decision making. There is currently a lack of a unified definition of clinical remission, limiting cross-study comparisons, and there should be a focus on future collaborative work in this area.
